# The role of social exclusion in the formation of suicidal ideation among the elderly in rural pension institutions

**DOI:** 10.3389/fpsyg.2022.1019898

**Published:** 2022-12-09

**Authors:** Pingda Wang, Peng Gao, Zehui Yu

**Affiliations:** ^1^School of Economics and Management, Northeast Agricultural University, Harbin, China; ^2^School of Business, China University of Political Science and Law, Beijing, China

**Keywords:** social exclusion, sense of belonging, depression, suicidal ideation, interpersonal trust

## Abstract

**Introduction:**

Social exclusion as well as a sense of belonging and depression have been identified as risk factors for suicide among older adults in pension institutions. In particular, the elderly living in rural pension institutions is more likely to have poor mental health and a higher incidence of suicidal ideation. This study explored the mechanism of social exclusion on suicidal ideation among the elderly in rural pension institutions, and the moderating effect of interpersonal trust.

**Methods:**

The social exclusion experience scale, sense of belonging, depression self-rating scale (CES-D), suicidal ideation scale (BSI-CV), and interpersonal trust scale (ITS) were used to investigate the elderly in rural pension institutions. A total of 1,387 samples (53.35% female) were collected, ranging in age from 65 to 95 years (M = 72.8, SD = 6.173).

**Results:**

The results of the study found that: (1) social exclusion increases the suicidal ideation of the elderly in rural pension institutions, and the sense of belonging and depression play a significant chain-mediated role in the relationship between social exclusion and suicidal ideation in the elderly. (2) Interpersonal trust moderates the impact of social exclusion on the sense of belonging, depression, and suicidal ideation. Specifically, interpersonal trust can alleviate the promotion effect of social isolation on suicidal ideation and depression, and can also reduce the adverse effect of social exclusion on the sense of belonging.

**Discussion:**

This study validates that social exclusion is a risk factor for suicidal ideation in the rural elderly and identifies interpersonal trust as a protective factor against social exclusion and its negative outcomes in the elderly. This study provides a scientific basis for improving the depression status of the elderly in rural China and formulating suicide prevention measures.

## Introduction

Suicide has become a serious global public health problem (Phillips, 2002). Every year, about 800,000 people die by suicide in the world, of which China accounts for about 17% of the total number, ranking second in the world (World Health Organization, 2019). Previous studies have shown that the group with the highest suicide rate is the elderly aged 65 years and above (Li et al., 2009; Wang et al., 2014). The results of China’s seventh census show that the aging process of China’s population has accelerated significantly and the urban and rural differences are significant (Bulletin of the Seventh National Population Census of the National Bureau of Statistics). Compared with urban areas, the rural population has a higher level of aging, and the mental health of the elderly is lower (Chen et al., 2018). At the same time, with the transformation of the traditional multi-generational family model in rural areas, the number of empty-nest families in rural areas is increasing day by day, and the proportion of rural pension institutions in the elderly care choices will be further expanded. However, whether in urban or rural areas, the incidence of suicidal ideation among the elderly in pension institutions is generally higher than that in the elderly living in the community (Mezuk et al., 2014). In particular, the elderly living in rural pension institutions are more likely to have poor mental health and a higher incidence of suicidal ideation (Tomasz et al., 2015; Zhang et al., 2017). In this context, the mental health and suicidal behavior of the elderly in rural pension institutions have become an important topic.

Numerous studies have examined associations between sociodemographic and socioenvironmental factors in older adults and suicidal ideation at the community level (Lester and Gatto, 1989; Ekramzadeh et al., 2012; Wu et al., 2010; Cohen-Louck and Aviad-Wilchek, 2020). However, social exclusion is usually the main factor leading to the decline of social relationship function in the elderly, and its important role in old age is seriously underestimated (Mezuk et al., 2014). Joiner’s interpersonal relationship theory of suicide also pointed out (Ribeiro and Joiner, 2009) that social exclusion is one of the most important causes of suicide, which can increase the individual’s suicidal ideation by reducing the individual’s perceived sense of belonging. Especially for the rural elderly with a small social range, the problem of social exclusion is even more prominent, because the elderly with experience of exclusion may not be able to find other alternative social relationships, and the resulting negative emotions will bring serious physical and mental health consequences (Frank et al., 2014; Eades et al., 2019). Importantly, those rural elderly who are socially excluded, they are likely to lose their sense of belonging to society, feel that they are separated from mainstream society, and eventually have a series of mental health problems (Feng et al., 2019). Empirical studies have shown that the most common negative emotion among socially excluded individuals is “depression” (Debono and Muraven, 2014; Wethington et al., 2016), and individuals with chronic depression will further develop into more severe conditions, such as self-harm and suicide and so on (Kim et al., 2018).

As an important social capital, interpersonal trust can not only help individuals cope with social exclusion and achieve the development of good interpersonal relationships, but also play an important role in promoting individual altruistic behavior, which is a key factor in suicide prevention (Righetti and Finkenauer, 2011). For example, the rural elderly with a higher level of interpersonal trust had less social exclusion experience on negative emotions (Liu et al., 2021). Because they believe that most people can be trusted, there will be a greater willingness and greater opportunity to repair the relationship with the rejecter (Decarli, 2003). Even if the relationship with the excluded cannot be repaired, the rural elderly with higher levels of interpersonal trust are more likely to have good social relationships with others (Demura and Mitsumori., 2013). Individuals will produce positive physiological and psychological responses through the establishment of social relationships, which not only make up for the lack of psychological needs but also effectively alleviate the negative emotions caused by social exclusion (Yip et al., 2007). That is to say, whether it is to repair the relationship with the excluded person or establish a new social relationship, the individual’s sense of belonging will be satisfied, and the depression caused by social exclusion will also be alleviated (Frank et al., 2014). In addition, other studies have also reported significant associations between social capital and suicidal behavior, and are thought to cure social factors that cause early suicidal ideation (Yamamura, 2010; You et al., 2011; Smith and Kawachi, 2014). Among them, interpersonal trust can play an important role in suicide prevention by keeping people away from the influence of suicidal ideation (Kim et al., 2017; Nie et al., 2020). Therefore, we can infer that interpersonal trust can be used as a protective factor for the elderly in rural pension institutions to help them effectively reduce the adverse effects of social exclusion on suicidal ideation.

Overall, although there is a strong relationship between social exclusion and suicidal ideation, the underlying processes that may mediate this relationship are largely unknown. Whether from the perspective of suicide prevention and control in the elderly or from the perspective of comprehensively achieving healthy aging, research on suicidal ideation in the elderly is a crucial topic. Therefore, in this particular era of increasing global population aging, it is crucial to explore the consequences of older people’s experiences of exclusion. Our research aims to investigate the mechanism of social exclusion on suicidal ideation among the elderly in rural pension institutions in China.

## Theoretical framework and research assumptions

### Social exclusion and suicidal ideation

Social exclusion refers to the erosion of social cohesion, the destruction of solidarity, and the lack of social integration (Silver, 1994), and it emphasizes that marginalized and disadvantaged groups in society are “excluded” from the mainstream and suffer from a variety of interrelated deprivation factors, preventing them from participating fully in society. In the field of psychology, social exclusion is a form that is common in all interpersonal relationships. Whether it is relatives, friends, or strangers, they may become the implementers and excluded persons of some forms of exclusion. Mental health and behavioral responses can have a huge impact.

Durkheim’s theory of suicide (Alpert et al., 1951) pointed out that social exclusion is one of the most important causes of suicide, and lack of social support and low social participation are important indicators of social exclusion. Suicidal ideation is an early stage of suicide, defined as active or passive thoughts of suicide at some point or stage in life (Waern et al., 1999; Dong et al., 2015), and is the strongest predictor of suicidal behavior (Nock et al., 2008). Numerous studies have shown that social exclusion is widespread in daily life (Williams, 2007) and occurs almost every day of our lives (Nezlek et al., 2012, 2015). For example, social exclusion in older adults is associated with factors such as mental and physical illness and suicide risk (Moak and Agrawal, 2009; Cacioppo et al., 2010), and experiences of exclusion can lead to greater dysfunction in older adults (Everard et al., 2000), pain (Dewall and Baumeister, 2006), and suicidal thoughts in later life (Waern et al., 2003; Wiktorsson et al., 2010). Other studies have found that subjects in the high social exclusion group have significantly higher suicidal ideation scores than the low social exclusion group. Individuals in the high social exclusion group will have more non-adaptive behaviors, and their negative psychological directly leads to negative behaviors such as self-harm and suicide (Nezlek et al., 1997; Debono and Muraven, 2014). Therefore, we have reason to believe that social exclusion will increase the suicidal ideation of the elderly in rural pension institutions. Accordingly, we propose the following assumption:

*H1*: Social exclusion increases the risk of suicidal ideation among older adults in rural pension institutions.

### The chain mediating effect of sense of belonging and depression

People are considered to have a strong sense of belonging and social interaction needs, and positive and sustainable social relationships are critical to people’s physical and mental health (Baumeister and Leary, 1997). However, the interpersonal interactions of individuals in everyday life are not always positive, social exclusion hinders people’s need for social relationships, belonging, and intimacy, it has a strong negative impact on the individual, and is a painful and sad experience (Williams, 2009; Nezlek et al., 2012). Social problems such as mental illness, aggression, and suicide caused by social exclusion have attracted the attention of scholars. They argue that social exclusion, as a negative aspect of interpersonal relationships, may show increasing levels of psychological distress and negative effects (Williams, 2007; Williams, 2009; Niu et al., 2016), as well as higher levels of Depression (Williams and Nida, 2011; Dewall et al., 2012). As pointed out by the Temporal need-threat model (Williams, 2007; Williams, 2009; Ren et al., 2013), when rejection persists, individuals feel a strong sense of insecurity, which eventually leads to suicidal behavior.

The sense of belonging of the elderly in the rural pension institution is the psychological feeling of satisfaction, recognition, love, and attachment of the elderly to the institution. Older adults who experience chronic social exclusion may experience a lower sense of belonging (Stillman et al., 2009). The interpersonal theory of suicide also proposes that social exclusion is a negative interpersonal experience, and the resulting low sense of belonging and perceived burden on others increases an individual’s risk of suicide (Van Orden et al., 2011). In addition, social exclusion can also induce negative emotions such as depression and loneliness, and lead to behavioral problems such as suicide. This is also confirmed by empirical research, namely, that social exclusion can lead to hampered needs such as belonging, a state of rapid decrease in positive emotions and an increase in negative emotions, making them feel depressed, depressed, and helpless, seriously impairing the mental health of older adults (Debono and Muraven, 2014; Wethington et al., 2016). The social exclusion experience of the elderly in rural pension institutions has a more significant negative effect on their physical and mental health (Hawton et al., 2011; Tong et al., 2011; Tong and Lai, 2016; Zhang et al., 2017), which will lead to Internalized psychological problems such as insomnia, depression and social pain, and then externalized behavioral problems such as aggression and suicide (Niu et al., 2016). In conclusion, belongingness and depression are mediating factors between social exclusion and suicidal ideation in the elderly, and belongingness can also reduce depression in the elderly. Based on this, we propose the following assumption:

*H2*: Belonging and depression play a chain mediating role between social exclusion and suicidal ideation in older adults.

### The moderating effect of interpersonal trust

Interpersonal trust is a general expectation that individuals believe that others or other groups are trustworthy, and it is the basis for establishing interpersonal relationships (Dekker et al., 2013), and plays an important role in maintaining interpersonal relationships and promoting interpersonal communication (Betts et al., 2009). There is growing evidence that interpersonal trust is associated with mental disorders and physical health. For example, a prospective study in the United States found that individuals with high levels of interpersonal trust were less likely to suffer from mental disorders (Fujiwara and Kawachi, 2008). Conversely, there was a positive relationship between distrust and poorer self-rated health, depression, and functional limitations (Pollack and Knesebeck, 2004). Compared with younger people, older adults are more likely to experience cognitive impairment and poor physical condition, which will lead to a greater need for the emotional support and material help typically found in trusting relationships, especially in the event of negative life events (Decarli, 2003; Demura and Mitsumori., 2013). The multiple motivational models of social exclusion state that individuals who are socially excluded will engage in prosocial behaviors (such as trying to repair social relations with the excluded person) and Anti-social responses (e.g., exhibiting aggressive behavior; Richman and Leary, 2009). Individuals with higher levels of interpersonal trust are more likely to engage in prosaically behaviors (Cadenhead and Richman, 1996) and produce positive physiological and psychological responses. Other studies have also confirmed this, that is, interpersonal trust is significantly positively correlated with mental health and social support, which can buffer the negative impact of risk factors on individual mental health (Frank et al., 2014). It can be seen that after being socially excluded from others or groups, the rural elderly with a higher level of interpersonal trust may have a stronger will and more opportunities to repair the relationship with the excluded person, produce more prosaically behaviors, and then have more prosocial behaviors. Satisfy the needs of inner belonging and relieve the negative emotions such as pressure, anxiety, and depression caused by social exclusion. On the contrary, after being socially excluded, the rural elderly with a low level of interpersonal trust may neither try to repair the relationship nor seek other alternative relationships due to the lack of trust in others, resulting in a lack of sense of belonging. And produce more intense depression, thereby increasing the possibility of suicide (Yu et al., 2019).

In addition, interpersonal trust also showed a strong association with the health and well-being of the rural Chinese population (Yip et al., 2007). The elderly in rural pension institutions in China have a smaller social circle, and interpersonal trust is crucial to their physical and mental health and well-being (Lu et al., 2011). They can obtain more social support through interpersonal trust, on the one hand, they can solve crisis of daily life, and negative life events, and adjust the bad psychological condition. On the other hand, it may also reduce the risk of mental disorders, suicidal ideation, and suicide attempts (Slater and Depue, 1981; Heikkinen et al., 1993).

Therefore, we can think that the lack of interpersonal trust will make the elderly in rural pension institutions unable to seek help and social support in time, leading to the lack of belonging and the deepening of psychological problems such as depression, and enhancing their suicidal ideation. Higher levels of interpersonal trust moderated the relationship between social exclusion and belonging, depression, and suicidal ideation.

*H3*: Interpersonal trust moderates the relationship between social exclusion and belonging.

*H4*: Interpersonal trust moderates the relationship between social exclusion and suicidal ideation.

*H5*: Interpersonal trust moderates the relationship between social exclusion and depression.

**Figure 1:**
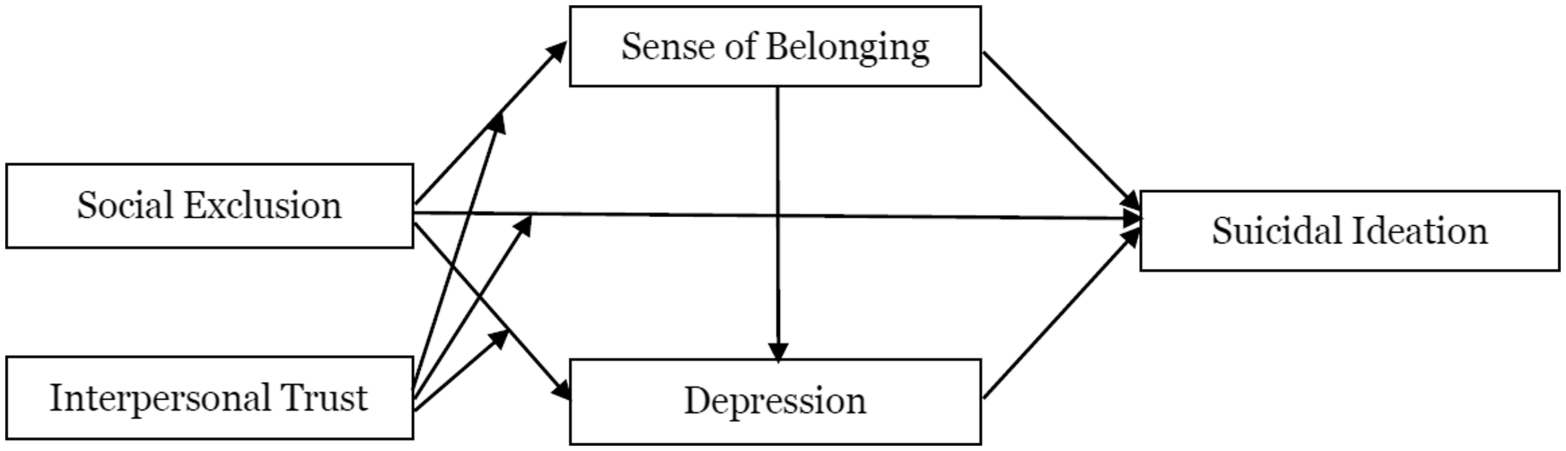
Research framework.

In summary, the research framework of this study is shown in Figure 1.

## Materials and methods

### Participants and procedures

All research procedures were approved by the ethics committee of the first author’s university and were conducted between November 2021 and May 2022. Seventeen undergraduate and graduate students majoring in psychology and social were trained as research assistants, who were primarily responsible for data collection after receiving unified training. According to the statistics of Heilongjiang Province 2021, there are nearly 1,000 existing registered nursing institutions in 12 cities, including Harbin, Qiqihar, Jixi, and Hegang, and rural nursing institutions account for about 80% of the total. The subjects of this study were selected based on the home locations of the research assistants (Harbin and Jixi). Through the preliminary communication with the heads of the institutions, 26 institutions were finally identified, taking into account the basic conditions such as the location of the elderly institutions, the number of people, the physical and mental conditions of the elderly and the willingness to participate. First of all, the research assistant will contact the potential elderly participants, and use unified instruction to explain the purpose of the questionnaire and the way of answering the questionnaire to the subjects to ensure that subjects fully understood the content of the questionnaire. For some elderly people with low education levels or with reading difficulties, the research assistants will answer the reading questions. After the questionnaire is completed, the investigators will confirm the completion of the filling on the spot, fill in the missing items in time, and re-verify the question. If the respondents fail to complete the missing items in time due to various reasons, they will be judged as invalid questionnaires. In this study, a total of 1,439 questionnaires were distributed, 1,391 questionnaires were returned, 4 incomplete questionnaires were excluded, and 1,387 valid questionnaires were finally obtained.

The age of the elderly ranged from 65 to 95 years old, with a mean of 72.80 years and a standard deviation of 6.173 years. Most of the elderly are 75 years old and below, accounting for 71.01%, and the elderly over 85 years old only accounted for 4.11%. The proportion of men and women in the sample is relatively balanced, of which 740 are women, accounting for 53.35%. The vast majority of the elderly have 5 or fewer children, accounting for 95.10%, and the number of elderly people with 2 and 3 children is the largest 435 (31.36%) and 405 (29.20%) respectively. 259 elderly people consider their health status to be poor, accounting for 18.67%, 739 elderly people consider their physical health status to be Fair, accounting for 53.3%, and elderly people think their physical health status is excellent, very good, and good are 14 (1.01%), 140 (10.09%) and 235 (16.94%) respectively. In addition, 145 (10.45%) elderly suffer from Diabetes, 32 (2.31%) elderly suffer from heart problem, 462 (33.31%) elderly suffer from Arthritis, and 144 (10.38%) elderly suffer from Dyslipidemia.

### Measures

#### Social exclusion

Using the Chinese version of the Social Exclusion Experience Scale (Liu et al., 2021) revised by Liu et al. (2021), the scale contains two dimensions of neglect and rejection, with a total of 8 items, such as: “When everyone talks together, I am often ignored by others,” “It seems that others often cannot see me”; the rejection entries are “When I appear, others often turn their backs,” “Others are always cold to me.” The scale is scored on a 7-point scale (1 = never, 7 = always), with higher scores indicating stronger experiences of social exclusion. In the present study, Cronbach’s alpha for the scale was 0.720.

#### Suicidal ideation

The Beck Suicidal Ideation Inventory-Chinese Version (BSI-CV) was compiled by Beck in 1979 based on clinical experience and theoretical research to measure the severity of suicidal ideation (Beck et al., 1979). The BSI-CV has a total of 19 items, and each item is scored on a three-level scale (0 moderate, 1 weak, 2 no), including “How much do you want to live?” “How much do you want to actively attempt suicide?” “You Questions such as how much you want to die,” measured the suicidal ideation of the subjects in the last week, and a higher total score means more serious suicidal ideation. In the present study, Cronbach’s alpha for the scale was 0.897.

#### Sense of belonging

The belongingness scale compiled by Cui Jie is used, which is widely used in Chinese groups (Li et al., 2017; Wang et al., 2019; Meng et al., 2022). The scale analyzes the sense of belonging of the elderly from three dimensions, including treating this pension institution as home, identifying with the institution, and being proud of this institution. There are a total of 10 questions, such as I do not think I belong to this pension institution, this pension institution makes you feel at home, etc. Each question is scored on a 5-point scale, ranging from completely agree (5 points) to completely disagree (1 point). Some items are scored in reverse, and the higher the score, the stronger the sense of belonging. In the present study, Cronbach’s alpha for the scale was 0.830.

#### Depression

The self-rating depression scale (Center for Epidemiological Survey, Depression Scale CES-D) compiled by Sirodff of the National Institute of Psychiatry in 1977 is used, with a total of 20 items, including (1) I am troubled by some small things; (2) I have trouble concentrating when doing things; (3) I feel down; (4) I find it hard to do anything, etc. The scale evaluation is based on the frequency of the corresponding situation or feeling in the past week; if it is less than 1 day, it is “none or basically absent”; 1–2 days is “rarely,” 3–4 days is “frequently,” and 5–7 days is “almost always.” The higher the score, the more severe the depression. In the present study, Cronbach’s alpha for the scale was 0.806.

#### Interpersonal trust

The Interpersonal Trust Scale (ITS) developed by Rotter in 1976 was used to measure the individual’s estimation of the reliability of others’ behavior and commitment (Rotter, 1967). The content includes interpersonal trust in various situations. Most items are related to the reliability of social roles, but some items are related to the degree of optimism about the future society. The scale contains 25 items, including items such as “There is more and more hypocrisy in our society” “The future seems promising” and “In this age of competition, others will take advantage of you if you are not vigilant.” In reverse question scoring, the scale is scored on a 5-point scale (1 = completely disagree, 5 = completely agree), and the higher the score, the higher the degree of interpersonal trust. The scale has good reliability and validity in Chinese subjects (Xin et al., 2013). In the present study, Cronbach’s alpha for the scale was 0.879.

### Statistical analysis

In this study, statistical analyses were conducted using SPSS 22.0. Data processing included the following steps. First, we described demographic variables and calculated correlation coefficients between the main variables. Nextly, we used the PROCESS macro for SPSS (Model 6) to test the chain mediating effect of sense of belonging and depression in the relationship between social exclusion on suicidal ideation (Hayes, 2013). Finally, we used the PROCESS macro for SPSS (Model 85) to investigate the moderating effect of interpersonal trust in the relationship between social exclusion on suicidal ideation, sense of belonging, and depression. Additionally, Age, gender, number of children, and education as control variables. All of the main variables were standardized before testing for the mediating and moderating effects.

## Results

### Preliminary analyses

The descriptive statistics and correlation coefficients were presented in Table 1. The results showed that elders who scored high levels of suicidal ideation were more likely to have high levels of social exclusion (*r* = 0.449, *p* < 0.01) and depression (*r* = 0.456, *p* < 0.01), and more likely to have low levels of sense of belonging (*r* = −0.405, *p* < 0.01) and interpersonal trust (*r* = −0.577, *p* < 0.01). Besides, interpersonal trust was negatively associated with social exclusion (*r* = −0.373, *p* < 0.01) and depression (*r* = −0.322, *p* < 0.01). In addition, sense of belonging was positively associated with interpersonal trust (*r* = 0.475, *p* < 0.01).

**Table 1:** Descriptive statistics and correlation analysis.

	Mean	SD	1	2	3	4	5
1. Suicidal Ideation	8.856	7.430	1				
2. Social Exclusion	18.949	4.336	0.449**	1			
3. Sense of Belonging	31.147	7.746	−0.405**	−0.316**	1		
4. Depression	48.442	8.207	0.456**	0.477**	−0.320**	1	
5. Interpersonal Trust	70.553	16.187	−0.577**	−0.373**	0.475**	−0.322**	1

**: Correlation is significant at the 0.01 level (2-tailed).

### Testing chain mediation effect of sense of belonging and depression

*Hypothesis 1* predicted that social exclusion increased the risk of suicidal ideation among older adults in rural pension institutions. *Hypothesis 2* predicted that sense of belonging and depression would play a chain mediating role in the relationship between social exclusion on suicidal ideation. We used Model 6 of the PROCESS macro to examine the above hypothesis (Hayes, 2013), and the results were presented in Table 2.

**Table 2:** Testing chain mediation effect of sense of belonging and depression.

	Model 1: Sense of Belonging		Model 2: Depression		Model 3: Suicidal Ideation	
	β	t	β	t	β	t
Social Exclusion	−0.299	−11.542***	0.406	16.416***	0.212	8.726***
Sense of Belonging			−0.181	−7.374***	−0.220	−9.786***
Depression					0.234	9.680***
Age	−0.008	−1.896	0.000	−0.008	−0.002	−0.624
Gender	−0.120	−2.154*	0.046	0.911	0.081	1.769
Number of children	0.068	3.570***	−0.049	−2.798**	−0.198	−12.645***
Education	−0.010	−0.680	−0.004	−0.325	−0.037	−3.027**
R^2^	0.113		0.264		0.407	
F	35.083***		82.554***		135.274***	

***p < 0.001; **p < 0.05; ***p < 0.01.

Model 1 in Table 2 showed that social exclusion was negatively associated with sense of belonging (*β* = −0.299, *p* < 0.001). Model 2 showed that social exclusion was positively associated with depression (*β* = 0.406, *p* < 0.001), and at the same time, sense of belonging was negatively associated with depression (*β* = −0.181, *p* < 0.001). Additionally, in model 3, social exclusion and depression were positively associated with suicidal ideation (β_Social Exclusion_ = 0. 212, *p* < 0.001; β_Depression_ = 0.234, *p* < 0.001), but sense of belonging was negatively associated with suicidal ideation (*β* = 0.-220, *p* < 0.001).

Therefore, it can be seen that both sense of belonging and depression are the mediator in the relationship between social exclusion and suicidal ideation, further, they were also a chain mediating in this relationship. The bootstrap 95% CI has confirmed this conclusion. As shown in Table 3, the 95% BootCI of each path does not contain zero, so the mediating and chain mediating effect were all significant. Additionally, Social Exclusion has a significant effect on suicidal ideation. So, *Hypothesis 2* was supported.

**Table 3:** Bias-corrected percentile bootstrap testing of each path.

Path	Effect	BootSE	95% Bootstap CI	
			Lower limit	Upper limit
X→Y	0.212	0.024	0.164	0.259
X→M1→Y	0.066	0.011	0.046	0.087
X→M2→Y	0.095	0.014	0.069	0.121
X→M1→M2→Y	0.013	0.003	0.007	0.019

X = Social Exclusion; Y=Suicidal Ideation; M1 = Sense of Belonging; M2 = Depression.

In model 3 shows a significant positively relationship between social exclusion and suicidal ideation, and we confirm this conclusion in the “X−Y” path of Table 3. So, *Hypothesis 1* was supported.

### Testing moderation effect of interpersonal trust

**Figure 2:**
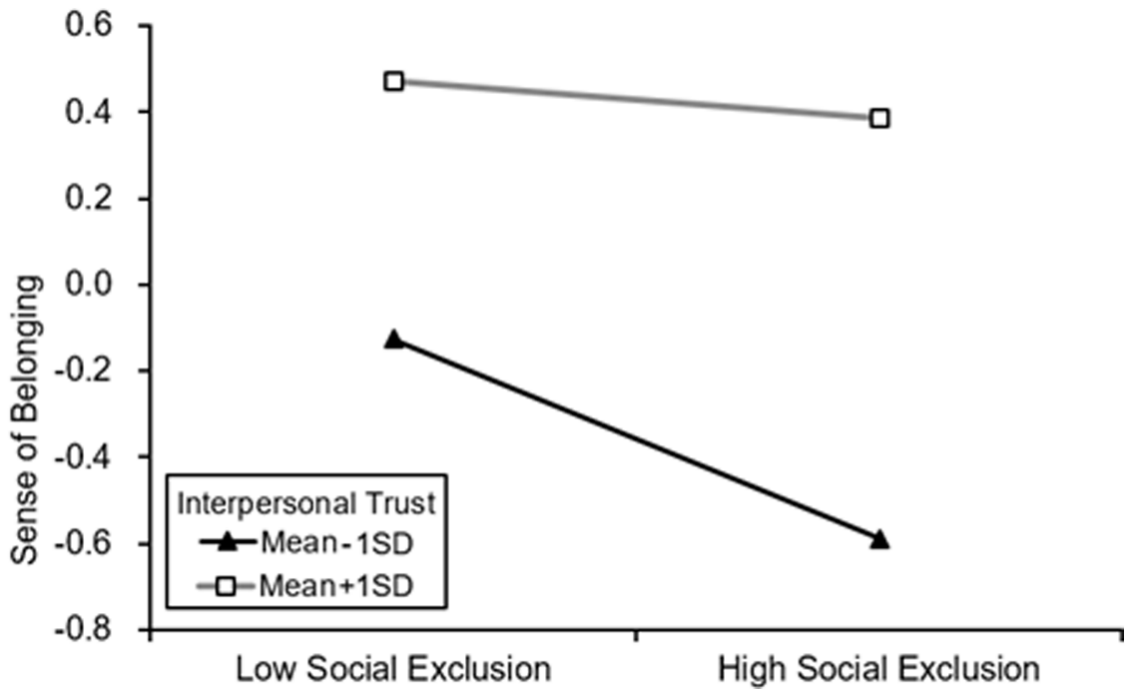
Interpersonal trust moderated the relationship between social exclusion on sense of belonging.

**Figure 3:**
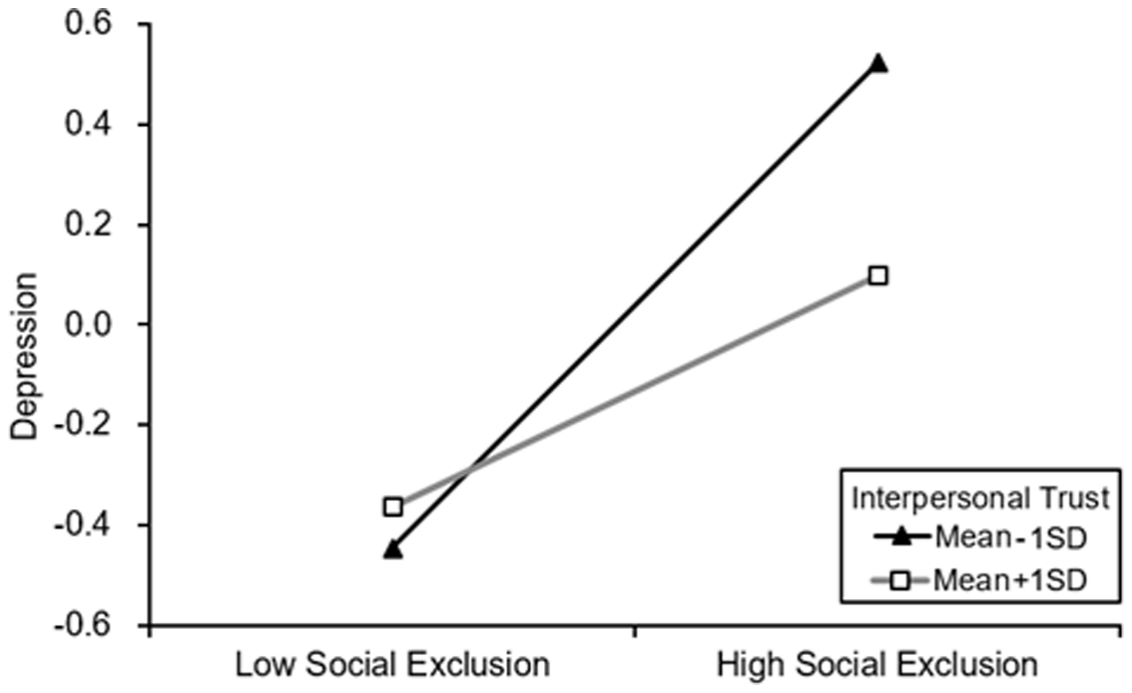
Interpersonal trust moderated the relationship between social exclusion on depression.

**Figure 4:**
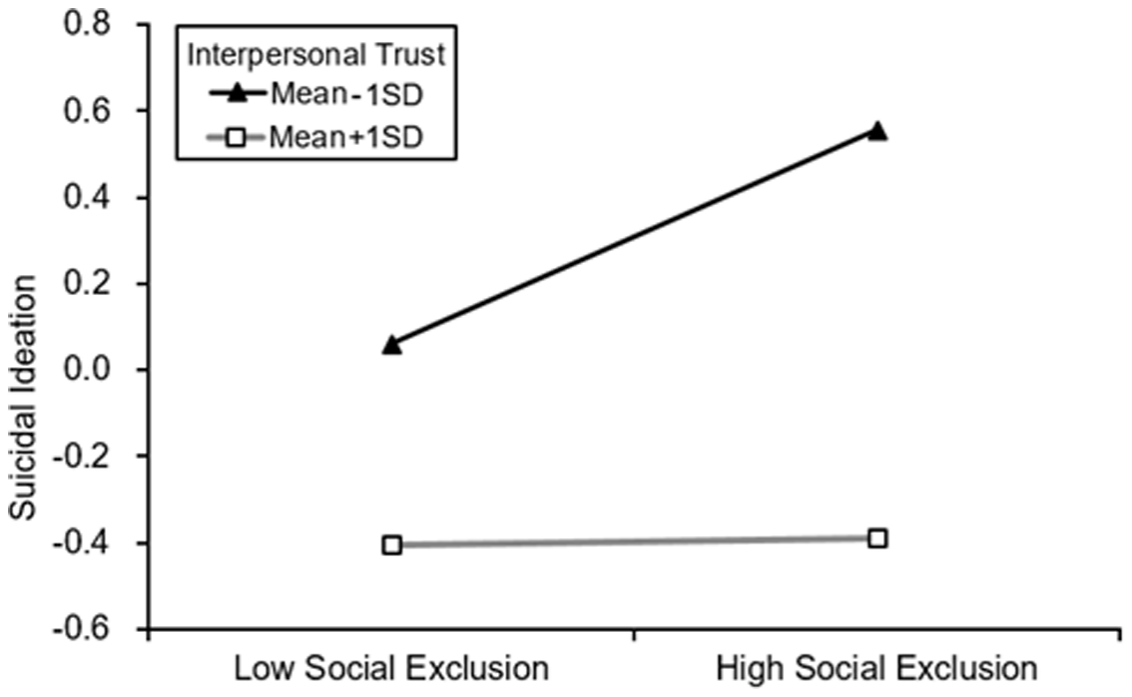
Interpersonal trust moderated the relationship between social exclusion on suicidal ideation.

*Hypothesis 3* ~ *Hypothesis 5* predicted that interpersonal trust would moderate the effect of social exclusion on sense of belonging, depression, and suicidal ideation. We used Model 85 of the PROCESS macro to examine the above hypothesis (Hayes, 2013), the results were presented in Table 4. And we also conducted simple slope tests to plot the results. Figures 2–4 showed the difference of influent in the relationship between social exclusion on sense of belonging, depression, and suicidal ideation when interpersonal trust at Mean + 1SD level and Mean-1SD level. In these figures, the Y-axis scale represents the values of this variable after standardization.

**Table 4:** Testing moderation effect of interpersonal trust.

	Model 1: Sense of Belonging		Model 2: Depression		Model 3: Suicidal Ideation	
	β	t	β	t	β	t
Social Exclusion	−0.137	−5.348 ***	0.357	14.052***	0.128	5.676***
Sense of Belonging			−0.124	−4.681***	−0.073	−3.318**
Depression					0.174	7.819***
Interpersonal Trust	0.393	15.343 ***	−0.086	−3.147**	−0.353	−15.612***
INT	0.094	4.622 ***	−0.127	−6.309***	−0.121	−7.141***
Age	−0.004	−1.031	−0.001	−0.277	−0.005	−1.502
Gender	−0.082	−1.615	0.036	0.733	0.066	1.590
Number of children	0.024	1.371	−0.052	−2.994**	−0.176	−12.131***
Education	−0.010	−0.741	−0.002	−0.180	−0.035	−3.182**
R2	0.262		0.291		0.515	
F	69.777***		70.735 ***		162.447***	

***p < 0.001; **p < 0.05; *p < 0.01.

INT = Interpersonal Trust × Social Exclusion.

Model 1 in Table 4 showed that social exclusion was negatively associated with sense of belonging (*β* = −0.137, *p* < 0.001), and the interaction term of social exclusion and interpersonal trust was positively related to sense of belonging as well (*β* = 0.094, *p* < 0.001). Therefore, the association between social exclusion and sense of belonging was moderated by interpersonal trust. Furthermore, it meant that for the elder who has a high level of interpersonal trust, the negative relationship between social exclusion and sense of belonging was weaker (β_higher_ = −0.043) than low level one (β_lower_ = −0.231), we plotted the above slope changes on Figure 2.

In addition, model 2 showed that social exclusion was positively associated with depression (*β* = 0.357, *p* < 0.001), however, the social exclusion and interpersonal trust’s interaction term was negatively related to depression (*β* = 0.094, *p* < 0.001). Therefore, the positively associated between social exclusion and depression was reduced by interpersonal trust. It meant that for the elder who has a high level of interpersonal trust, the positive relationship between social exclusion and depression was weaker (β_higher_ = 0.230) than low level one (β_lower_ = 0.484), we plotted the above slope changes in Figure 3.

Finally, the model 3 showed that social exclusion was positively associated with suicidal ideation (*β* = 0.128, *p* < 0.001), but the interaction term (social exclusion× interpersonal trust) was negatively related to suicidal ideation (*β* = 0.094, *p* < 0.001). Therefore, the association between social exclusion and sense of belonging was moderated by interpersonal trust can relieve the promoting effect of social isolation on suicidal ideation. For the elder who has a high level of interpersonal trust, the positive relationship between social exclusion and suicidal ideation was weaker (β_higher_ = 0.007) than low level one (β_lower_ = 0.248), we plotted the above slope changes in Figure 4.

Above all, *Hypothesis 3* ~ *Hypothesis 5* were supported.

## Discussion

There is growing empirical support for the adverse effects of social exclusion on suicidal ideation. However, the mediating and moderating mechanisms behind this association remain largely unexplored, especially for the elderly in rural pension institutions. To explore this mechanism, this study used a sample of 1,387 elderly questionnaires in rural pension institutions to examine the chain mediating effect of sense of belonging and depression and the moderating effect of interpersonal trust. The results show that social exclusion affects suicidal ideation in older adults by reducing their sense of belonging and increasing their depression, and the lack of sense of belonging also increases the likelihood of depression. More importantly, findings from the current moderated mediation model suggest that interpersonal trust partially moderates the association between social exclusion and suicidal ideation. Overall, this study revealed that social exclusion is widespread in rural pension institutions and is the main reason for the formation of suicidal ideation in the elderly. Therefore, the mental health problems of the elderly in rural pension institutions still require continuous and continuous attention, which can help to develop targeted prevention and intervention plans to improve the coping ability of the elderly when they experience social exclusion.

First, our study showed that there is a positive relationship between social exclusion and suicidal ideation, that is, older adults who experience social exclusion have higher levels of suicidal ideation (Waern et al., 1999). In China’s traditional agricultural society, the elderly enjoy prestige and respect in rural households (Dong et al., 2014). However, in recent years, more and more rural youth are working outside the home, and coupled with the shortage of family caregivers who can provide long-term home care for the elderly, many elderly people have to live in pension institutions (Yang and Ou, 2013). This makes the elderly fall into a serious social, family, and personal crisis, and gradually become a vulnerable group in society and are “excluded” from the mainstream. In this case, if the elderly lack a certain amount of social support, they may be more vulnerable to stress in all aspects of life, resulting in suicidal thoughts (Yu et al., 2019).

Secondly, the results of the study found that belonging and depression play a chain mediating role between social exclusion and suicidal ideation in rural elderly. The sense of belonging of the elderly in the rural pension institution emphasizes their psychological feelings of satisfaction, identification, love, and attachment to the rural pension institution (Zhang, 2013), while the rural elderly who are socially excluded will have a series of problems due to the inability to satisfy their psychological sense of belonging. Negative emotions, which in turn increase suicidal ideation in the elderly. Because social exclusion or denial by social groups not only hinders the need to belong, but also reduces the relational value between individuals, and this often causes intense anxiety (too potential rejection) and depression (too actual rejection), and other negative emotions (Leary Mark, 1990; Sun et al., 2020), ultimately leading to adverse cognitive, emotional and physical effects on the individual (Pickett et al., 2004; Baumeister et al., 2005). Even excluded individuals will experience a significant increase in self-defeating behavior due to cognitive disintegration (Twenge et al., 2007). As indicated by the multi-motivation model of social exclusion (Richman and Leary, 2009), for the rural elderly who experience social exclusion, the lack of belonging and depression caused by social exclusion are key factors leading to suicidal ideation. In China, the concepts of “falling leaves return to their roots” and “raising children to prevent old age” are deeply rooted, and the elderly in rural areas seldom choose to live in rural pension institutions. However, they often have to live in rural pension institutions due to reasons such as female migrant workers or a decline in their ability to take care of themselves (Zi-Wei et al., 2019). This means that the elderly will leave the original family environment and face the pressure of adapting to the new environment. In addition, the inability to take care of themselves and their disability will weaken the ability of the elderly to participate in social activities and social interactions, increase their sense of social isolation, and make rural pension institutions. The elderly are more prone to psychological distress such as loneliness, depression, and even suicidal ideation (Zhang et al., 2017). In addition, some elderly people cannot receive home care due to physical dysfunction, which is the most common reason for staying in pension institutions. This group of older adults may be more vulnerable to exclusion from others, which can lead to the onset and exacerbation of depression and an increased risk of suicide (Stegenga et al., 2012; Fässberg et al., 2015).

Finally, based on the above results, it can be seen that social exclusion is a common phenomenon of social connection destruction for individuals, which will lead to physical and psychological pain in individuals, but interpersonal trust can effectively alleviate the negative effects of social exclusion. Our findings found that interpersonal trust moderates the effects of social exclusion on suicidal ideation, belonging, and depression. Specifically, interpersonal trust can moderate the promoting effects of social isolation on suicidal ideation and depression, while also reducing the adverse effects of social exclusion on belonging. Elderly people with higher interpersonal trust are more likely to establish stable and harmonious interpersonal relationships within a smaller range (usually in pension institutions), thereby defusing the harm caused by social exclusion. It can also be said that interpersonal trust is associated with better social functioning, physical and mental health, and the development of interpersonal relationships (Cadenhead and Richman, 1996; Frank et al., 2014). It can not only meet the needs of the elderly’s sense of belonging, but also enhance the individual’s ability to adapt to the environment and communication skills, help the elderly to reveal their inner emotions and feelings, and reduce the risk of depression and suicide. In addition, another study also confirmed the strong association between interpersonal trust and the health and well-being of the Chinese rural population (Lu et al., 2011). In the current context of China, the elderly in rural pension institutions have a smaller social scope, and the level of interpersonal trust is crucial to their physical and mental health and life well-being. They can gain more social support through interpersonal trust. On the one hand, it can solve the crisis of daily life, and negative life events, and adjust the bad psychological condition. On the other hand, it may reduce the risk of mental disorders, and reduce suicidal ideation and suicide attempts. Therefore, it is necessary to prevent the effect of social exclusion in daily life on the suicidal ideation of elderly people by enhancing the interpersonal trust of elderly people in pension institutions. Considering the weakened status, physical function, and social role of elderly people in pension institutions, they are prone to encounter the dilemma of insufficient resources in rural pension institutions. It is suggested that the government level can improve the supply of public services to rural elderly institutions, which may be beneficial to enhance the social inclusion and social trust of the elderly (Lu and Zhang, 2014). Therefore, there is a need to enhance policy interventions in this regard. In addition, the positive impact of personal well-being on the interpersonal trust of older adults is considered. In rural institutions, older adults who are happy with their situation are more likely to transmit this positive mindset to other older adults. Therefore, enhancing the personal well-being of older adults is of great practical importance in promoting their interpersonal trust.

Overall, this study validated social exclusion as a risk factor for suicidal ideation in rural older adults and identified interpersonal trust as a protective factor for social exclusion in older adults and its cascade of consequences such as depression and suicidal ideation. To provide a scientific basis for depression status and the development of suicide prevention measures among the elderly in rural China.

## Conclusion

This study shows that social exclusion was positively associated with suicidal ideation among older adults in rural pension institutions while belonging and depression play a chain-mediated mediating role in this relationship. Interpersonal trust levels in older adults moderate the adverse effects of social exclusion on belonging, depression, and suicidal ideation. Our study examines the social exclusion status of the elderly in rural pension institutions and its impact mechanism on suicidal ideation and provides empirical support for reducing the risk of suicide in the elderly. We hope that this study will draw scholars’ attention to the elderly in pension institutions. Because interpersonal relationships are essential to health and quality of life at all ages, they may be a particularly useful target for interventions for older adults to promote their mental health in later life. Although declines in physical, sensory, and cognitive function in older adults are associated with suicidal ideation, interpersonal trust remains malleable throughout life. Although rural pension institutions can provide professional and timely life care to the elderly and meet their basic physiological needs, they still need to be further strengthened in terms of providing psychological counseling and emotional support.

### Limitations and further research directions

This study also has some shortcomings. First, social exclusion can affect health status, but at the same time, individuals with lower health status may also be more vulnerable to exclusion from others (Sacker et al., 2017). Future research can further verify the relationship between social exclusion and health status and its psychological mechanism by collecting longitudinal data. Second, although existing studies analyzing suicide all mention suicide rates, there appears to be a large gap between suicidal behavior and suicidal ideation. It should be noted that there may be information bias when using suicidal ideation rather than completed suicide as the outcome variable. For example, some people are thought to exhibit greater suicidal thoughts, even though they are not planning to kill themselves. Therefore, differences in outcomes between suicidal ideation and suicide may reflect information bias. In future studies, this difference will be examined. Finally, the current study addresses only suicidal ideation due to social exclusion. To effectively prevent suicide in older adults, it is worth investigating how and why individuals consider suicide, even if they do not commit suicide. Therefore, further research is needed to better understand the background of the elderly in rural pension institutions and to examine the underlying factors that contribute to their suicidal ideation.

## Data availability statement

The original contributions presented in the study are included in the article/supplementary material; further inquiries can be directed to the corresponding author.

## Ethics statement

The studies involving human participants were reviewed and approved by Ethics Committee of Northeast Agricultural University. The patients/participants provided their written informed consent to participate in this study.

## Author contributions

PW is the experimental designer and executor of this study. PG is the designer and person in charge of the project, guiding the writing and modification of the experimental design data analysis paper. ZY have completed data analysis and written the first draft of the paper. All authors contributed to the article and approved the submitted version.

## Funding

This work was supported by Heilongjiang Philosophy and Social Science Research Project (14B007), Humanities and Social Science Research Project of The Provincial Department of Education (12542008), Chongqing Construction Science and Technology Plan Project (2022[6]-2), and the Science and Technology Research Program of Chongqing Municipal Education Commission (Grant No. KJQN202100811).

## Conflict of interest

The authors declare that the research was conducted in the absence of any commercial or financial relationships that could be construed as a potential conflict of interest.

## Publisher’s note

All claims expressed in this article are solely those of the authors and do not necessarily represent those of their affiliated organizations, or those of the publisher, the editors and the reviewers. Any product that may be evaluated in this article, or claim that may be made by its manufacturer, is not guaranteed or endorsed by the publisher.
